# Prediction of Functional Class of Proteins and Peptides Irrespective of Sequence Homology by Support Vector Machines

**DOI:** 10.4137/bbi.s315

**Published:** 2009-11-24

**Authors:** Zhi Qun Tang, Hong Huang Lin, Hai Lei Zhang, Lian Yi Han, Xin Chen, Yu Zong Chen

**Affiliations:** 1 Department of Pharmacy and Department of Computational Science, National University of Singapore, Republic of Singapore, 117543; 2 Department of Biotechnology, Zhejiang University, Hang Zhou, Zhejiang Province, P. R. China, 310029; 3 Shanghai Center for Bioinformatics Technology, Shanghai, P. R. China, 201203

**Keywords:** machine learning method, peptide function, protein family, protein function, protein function prediction, support vector machines

## Abstract

Various computational methods have been used for the prediction of protein and peptide function based on their sequences. A particular challenge is to derive functional properties from sequences that show low or no homology to proteins of known function. Recently, a machine learning method, support vector machines (SVM), have been explored for predicting functional class of proteins and peptides from amino acid sequence derived properties independent of sequence similarity, which have shown promising potential for a wide spectrum of protein and peptide classes including some of the low- and non-homologous proteins. This method can thus be explored as a potential tool to complement alignment-based, clustering-based, and structure-based methods for predicting protein function. This article reviews the strategies, current progresses, and underlying difficulties in using SVM for predicting the functional class of proteins. The relevant software and web-servers are described. The reported prediction performances in the application of these methods are also presented.

## Introduction

Functional clues contained in the amino acid sequence of proteins and peptides (Bork et al. 1998; Eisenberg et al. 2000; Bock and Gough, 2001; Lo et al. 2005) have been extensively explored for computer prediction of protein function and functional peptides. Sequence similarity (Baxevanis, 1998; Bork and Koonin, 1998; Schuler, 1998), motifs (Hodges and Tsai, 2002), clustering (Enright and Ouzounis, 2000; Enright et al. 2002; Fujiwara and Asogawa, 2002), and evolutionary relationships (Eisen, 1998; Benner et al. 2000) are typical examples of highly successful methods for facilitating functional prediction of proteins and peptides, which are primarily based on some form of sequence similarity or clustering. However, these methods tend to become less effective in the absence of sufficiently clear sequence similarities (Eisen, 1998; Rost, 2002; Whisstock and Lesk, 2003). In a comprehensive evaluation of sequence alignment methods against 15,208 enzymes labeled with an International Enzyme Commission EC class index, it has been found that approximately 60% of the EC classes containing two or more enzymes could not be perfectly discriminated by sequence similarity at any threshold (Shah and Hunter, 1997). The low and non-homologous proteins of unknown function constitute a substantial percentage, up to 20%~100%, of the open reading frames (ORFs) in many of the currently completed genomes (Han et al. 2004a). Therefore, it is desirable to explore other methods that are less dependent or independent of sequence or structural similarity (Smith and Zhang, 1997; Eisenberg et al. 2000).

In the last few years, there have been significant progresses in the development of alternative functional prediction methods to reduce the dependence on sequence similarity and clustering. For instance, non-sequence features such as structural features (Teichmann et al. 2001; Todd et al. 2001), interaction profiles (Aravind, 2000; Bock and Gough, 2001), and protein/gene fusion data (Enright et al. 1999; Marcotte et al. 1999) have been used for predicting protein functions. Machine learning methods have been explored for predicting protein function from amino acid sequence derived structural and physicochemical properties (des Jardins et al. 1997; Jensen et al. 2002; Karchin et al. 2002; Jensen et al. 2003; Cai et al. 2003; Cai and Lin, 2003; Cai et al. 2004b; Bhasin and Raghava, 2004a; Han et al. 2004b; Cai and Chou, 2005; Guo et al. 2006). In particular, one of the machine learning methods, support vector machines (SVM), have shown promising potential for predicting proteins and peptides of various biochemical classes (ae.g. receptors (Bhasin and Raghava, 2004a; Bhasin and Raghava, 2004b; Yabuki et al. 2005), nucleic acid or lipid binding proteins (Cai and Lin, 2003; Bhardwaj et al. 2005; Guo et al. 2006; Lin et al. 2006c), enzymes (Cai et al. 2004b; Cai and Chou, 2005; Dobson and Doig, 2005)), therapeutic groups (e.g. hormone proteins (Jensen et al. 2003), stress response proteins (Jensen et al. 2003), cytokines (Huang et al. 2005), MHC-binding peptides (Bhasin and Raghava, 2004c)), and other broadly defined functional classes (e.g. crystallizable proteins (Smialowski et al. 2006), mitochondrial proteins (Kumar et al. 2006), and functional classes in yeast (Cai and Doig, 2004)).

This article reviews the strategies, performances, current progresses and difficulties in applying SVM for predicting various functional classes and interaction profiles of proteins and peptides. Algorithms for representing proteins and peptides by using amino acid sequence derived structural and physicochemical descriptors (Bock and Gough, 2001; Karchin et al. 2002; Cai et al. 2003; Gasteiger, 2005) are also discussed. Web servers for facilitating the computation of these descriptors and for predicting the functional classes of proteins and peptides by the SVM method are discussed.

## Functional Classes of Proteins and Peptides

Apart from sequence and structural classes, proteins have been classified into functional classes. Active sites of the members of each class share common structural and physicochemical properties to support the common functionality, which can be explored for predicting the function of proteins from amino acid sequence derived structural and physicochemical descriptors independent of sequence homology. One example is enzyme families. Enzymes represent the largest and most diverse group of all proteins, catalyzing chemical reactions in the metabolism of all organisms. Based on their catalyzed chemical reactions, enzymes can be divided into three levels of functional classes. The first level is composed of 6 super families (EC1 oxidoreductases, EC2 transferases, EC3 hydrolases, EC4 lyases, EC5 isomerases, and EC6 ligases), the second level contains 63 families (such as EC3.4 hydrolases acting on peptide bonds and EC4.1 carbon-carbon lyases), and the third level contains 254 subfamilies (such as EC2.7.1 phosphotransferases with an alcohol group as acceptor). Active sites of enzymes are inherently reactive environments packed with specific types of amino acid residues and cofactors, and these and other structural features facilitate binding and catalysis of specific types of substrates (Cai et al. 2004b).

Another example is DNA binding proteins, which play critical roles in regulating such genetic activities as gene transcription, DNA replication, DNA packaging, and DNA repair (Lewin, 2000). Prediction of DNA-binding proteins is important for studying proteins involved in genetic regulation (Aguilar et al. 2002; Stawiski et al. 2003; Sarai and Kono, 2005). DNA recognition by proteins is primarily mediated by combination of such structural and physicochemical features as specific DNA binding domains (Bewley et al. 1998; Garvie and Wolberger, 2001), helix structures (Garvie and Wolberger, 2001), minor groove binding architectures (Bewley et al. 1998), asymmetric phosphate charge neutralization (Bewley et al. 1998), conserved amino acids (Luscombe and Thornton, 2002), hydrogen bonds (Luscombe et al. 2001), water-mediated bonds (Fujii et al. 2000; Luscombe et al. 2001), and indirect recognition mechanism (Steffen et al. 2002). DNA-binding proteins can be further divided into 9 major functional classes plus several smaller ones (such as covalent protein-DNA linkage proteins and terminal addition proteins). The 9 major classes are DNA condensation (for wrapping of DNA around histones), DNA integration (mediating the insertion of duplex DNA into a chromosome), DNA recombination (for cleaving and rejoining DNA), DNA repair, DNA replication, DNA-directed DNA polymerase (catalyzing DNA synthesis by adding deoxyribonucleotide units to a DNA chain using DNA as a template), DNA-directed RNA polymerase (catalyzing RNA synthesis by adding ribonucleotide units to a RNA chain using DNA as a template), repressor (interfering with transcription by binding to specific sites on DNA), and transcription factor.

The third example is transporter families. Transporters play key roles in transporting cellular molecules across cell and cellular compartment boundaries, mediating the absorption and removal of various molecules, and regulating the concentration of metabolites and ionic species (Hediger, 1994; Seal and Amara, 1999; Borst and Elferink, 2002). Specific transporters have been explored as therapeutic targets (Dutta et al. 2003; Joet et al. 2003; Birch et al. 2004) and a variety of transporters are responsible for the absorption, distribution and excretion of drugs (Kunta and Sinko, 2004; Lee and Kim, 2004). Thus functional assignment of transporters is important for facilitating drug discovery and research of genomics, cellular processes and diseases. There are active and passive transporters. Active transporters couple solute transport to the input of energy and these can be divided into two classes: ion-coupled and ATP-dependent transporters. Ion-coupled transporters link uphill solute transport to downhill electrochemical ion gradients. ATP-dependent transporters are directly energized by the hydrolysis of ATP and they transport a heterogeneous set of substrates. Passive transporters include facilitated transporters and channels, which allow the diffusion of solutes across membranes. These transporters evolve from common themes into families of different architectures (Hediger, 1994; Driessen et al. 2000; Saier, 2000). Transporters are divided into TC families based on their mode of transport, energy coupling mechanism, molecular phylogeny and substrate specificity (Saier, 2000). TC families are classified at four levels (TC class, TC sub-class, TC family, and TC sub-family) as indicated by a specific TC number TC I.X.J.K.L. Here I = 1, …, 9 represents each of the 9 TC classes, X = A, B, C, D, E, … represents each of the TC sub-classes that belong to a TC class, J = 1, … represents each of the TC families that belong to a TC sub-class, K = 1, … represents each of the TC sub-families that belong to a TC family, and L = 1, … represents individual transporters under a sub-family.

The fourth example is lipid-binding proteins, which play important roles in cell signaling and membrane trafficking (Downes et al. 2005), lipid metabolism and transport (Glatz et al. 2002; Haunerland and Spener, 2004), innate immune response to bacterial infections (Bingle and Craven, 2004), and regulation of gene expression and cell growth (Bernlohr et al. 1997). Prediction of the functional roles of lipid-binding proteins is important for facilitating the study of various biological processes and the search of new therapeutic targets. Lipid-binding proteins are diverse in sequence, structure, and function (Niggli, 2001; Pebay-Peyroula and Rosenbusch, 2001; Hanhoff et al. 2002; Weisiger, 2002; Bolanos-Garcia and Miguel, 2003; Palsdottir and Hunte, 2004; Fyfe et al. 2005; Balla 2005). Non-the-less, lipid recognition by proteins is primarily mediated by some combination of a number of structural and physicochemical features including conserved fold elements (Bernlohr et al. 1997), specific lipid-binding site architectures (Niggli, 2001) and recognition motifs (Palsdottir and Hunte, 2004; Balla, 2005), ordered hydrophobic and polar contacts between lipid and protein (Pebay-Peyroula and Rosenbusch, 2001), and multiple noncovalent interactions from protein residues to lipid head groups and hydrophobic tails (Palsdottir and Hunte, 2004). There are 8 major lipid-binding classes, which include lipid degradation, lipid metabolism, lipid synthesis, lipid transport, lipid-binding, lipopolysaccharide biosynthesis, lipoprotein (proteins posttranslationally modified by the attachment of at least one lipid or fatty acid, e.g. farnesyl, palmitate and myristate), lipoyl (proteins containing at least one lipoyl-binding domain).

One of the intensively studied peptide classes is MHC-binding peptides (Bhasin and Raghava, 2004c). Peptide binding to MHC is critical for antigen recognition by T-cells. One of the mechanisms of immune response to foreign or self protein antigens is the activation of T-cells by the recognition of T-cell receptors of specific peptides degraded from these proteins and transported to the surface of antigen presenting cells (Abbas and Lichtman, 2005). Peptides recognized by T-cells are potential tools for diagnosis and vaccines for immunotherapy of infectious, autoimmune, and cancer diseases (Shoshan and Admon, 2004). In many respects, MHC-binding and other protein-binding peptides possess similar characteristics as proteins of specific functional classes in that they also share some structural and physicochemical features to facilitate the common function: binding to MHC or other proteins (Matsumura et al. 1992; Zhang et al. 1998; McFarland and Beeson, 2002).

## Support Vector Machine Approach for Predicting Functional Classes of Proteins and Peptides

Support vector machines can be explored for functional study of proteins and peptides by determining whether their amino acid sequence derived properties conform to those of known proteins and peptides of a specific functional class (Cai and Lin, 2003; Cai et al. 2004b; Cai and Doig, 2004; Han et al. 2004b; Dobson and Doig, 2005).

The advantage of this approach is that more generalized sequence-independent characteristics can be extracted from the sequence derived structural and physicochemical properties of the multiple samples that share common functional or interaction profiles irrespective of sequence similarity. These properties can be used to derive classifiers (Bock and Gough, 2001; Bock and Gough, 2003; Cai and Lin, 2003; Han et al. 2004b; Xue et al. 2004b; Bhasin and Raghava, 2004c; Cai et al. 2004b; Cai and Doig, 2004; Dobson and Doig, 2005; Lo et al. 2005; Martin et al. 2005; Ben-Hur and Noble, 2005) for predicting other proteins and peptides that have the same functional or interaction profiles.

The task of predicting the functional class of a protein or peptide can be considered as a two-class (positive class and negative class) classification problem for separating members (positive class) and non-members (negative class) of a functional or interaction class. SVM and other well established two-class classification-based machine learning methods can then be applied for developing an artificial intelligence system to classify a new protein or peptide into the member or non-member class, which is predicted to have a functional or interaction profile if it is classified as a member. Sequence-derived structural and physicochemical properties have frequently been used for representing proteins and peptides (Bock and Gough, 2001; Bock and Gough, 2003; Cai and Lin, 2003; Bhasin and Raghava, 2004c; Cai et al. 2004b; Cai and Doig, 2004; Han et al. 2004b; Ben-Hur and Noble, 2005; Dobson and Doig, 2005; Lo et al. 2005; Martin et al. 2005) in the development of SVM and other machine learning classification systems for predicting the functional and interaction profiles of proteins.

**Figure 1.**
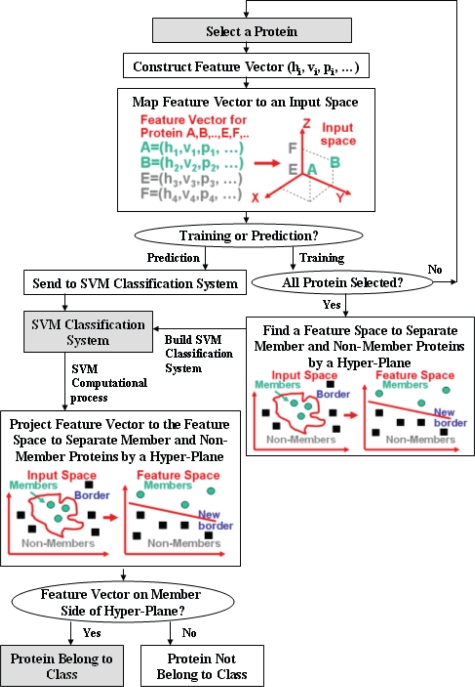
Schematic diagram illustrating the process of the training and prediction of the functional class of proteins and peptides by using support vector machine (SVM) method. A,B: feature vectors of proteins belong to a functional class; E,F: feature vectors of proteins not belong to a functional class. Sequence-derived feature h_j_, v_j_, p_j_, … represents such structural and physicochemical properties as hydrophobicity, polarizability, and volume; or such properties as domain information, subcellular localization, and post-translational (PT) modification profiles etc.

Figure 1 illustrates the process of using SVM for training and predicting proteins or peptides that have a specific common functional or interaction profile. Proteins or peptides known to have and not have the profile are represented by separate sets of feature vectors, which are composed of descriptors derived from the sequence of these proteins or peptides for representing their structural and physicochemical properties. These two sets of feature vectors are projected into a multi-dimensional space in which they are separated by a hyper-plane in such a way that those having the profile are on one side and those without the profile are on the other side of the hyper-plane. A new protein or peptide can be predicted to have the same profile if its feature vector is projected on the side of the hyper-plane where other proteins or peptides having the profile are located.

## Representation of Protein and Peptide Sequences

Protein or peptide sequences have been represented by a number of amino acid sequence derived structural and physicochemical descriptors (Bock and Gough, 2001; Karchin et al. 2002; Cai et al. 2003; Gasteiger, 2005). They include amino acid composition, dipeptide composition, sequence autocorrelation descriptors, sequence coupling descriptors, and the descriptors for the composition, transition and distribution of hydrophibicity, polarity, polarizibility, charge, secondary structures, and normalized Van der Waals volumes. Web servers such as PROFEAT (Li et al. 2006) (http://jing.cz3.nus.edu.sg/cgi-bin/prof/prof.cgi) and ProtParam (Gasteiger et al. 2005) (http://www.expasy.org/tools/protparam.html) have appeared for facilitating the computation of these descriptors. CBS Prediction Servers (http://www.cbs.dtu.dk/services/) can be used for computing other sequence derived features such as cleavage sites, nuclear export signals, and subcellular localization.

Amino acid composition is the fraction of each amino acid type in a sequence *f* (*r*) = *N_r_* */ N*, where r = 1, 2, 3, …, 20, N_r_ is the number of amino acid of type r and N is sequence length. Dipeptide composition is defined as *fr* (*r,s*) = *N_rs_ / (N–*1), where r,s = 1, 2, 3, …, 20, and N_ij_ is the number of dipeptide represented by amino acid type r and s (Bhasin and Raghava, 2004a). Autocorrelation descriptors are defined from the distribution of amino acid properties along the sequence (Kawashima and Kanehisa, 2000). The amino acid indices used in these autocorrelation descriptors include hydrophobicity scales (Cid et al. 1992), average flexibility indices (Bhaskaran and Ponnuswammy, 1988), polarizability parameter (Charton and Charton, 1982), free energy of solution in water (Charton and Charton, 1982), residue accessible surface area in trepeptide (Chothia, 1976), residue volume (Bigelow, 1967), steric parameter (Charton, 1981), and relative mutability (Dayhoff and Calderone, 1978). Each of these indices is centralized and normalized before the calculation. The frequently used autocorrelated descriptors include Moreau-Broto autocorrelation descriptors, normalized Moreau-Broto autocorrelation descriptors and Geary autocorrelation descriptors.

The quasi-sequence-order descriptors are derived from both the Schneider-Wrede physicochemical distance matrix (Schneider and Wrede, 1994; Chou, 2000; Chou and Cai, 2004) and the Grantham chemical distance matrix (Grantham, 1974) between the 20 amino acids.

Three descriptors, composition (C), transition (T) and distribution (D), are derived for each of the following physicochemical properties: hydrophibicity, polarity, polarizibility, charge, secondary structures, and normalized Van der Waals volume (Dubchak et al. 1995; Dubchak et al. 1999; Cai et al. 2003). For each property, the constituent amino acids in a protein or peptide are divided in three classes according to its attribute such that each amino acid is encoded by one of the indices 1, 2, 3 according to the class it belongs to. For instance, amino acids can be divided into hydrophobic (CVLIMFW), neutral (GASTPHY), and polar (RKEDQN) groups. C represents the number of amino acids of a particular property (such as hydrophobicity) divided by the total number of amino acids in a protein sequence. T characterizes the percent frequency with which amino acids of a particular property is followed by amino acids of a different property. D measures the chain length within which the first, 25%, 50%, 75% and 100% of the amino acids of a particular property is located respectively. Overall, there are 21 elements representing these three descriptors: 3 for C, 3 for T and 15 for D.

## Algorithms and Software Tools of Support Vector Machines

SVM can be divided into linear and nonlinear SVM. Linear SVM directly constructs a hyperplane in the feature space to separate positive examples from negative examples. On the other hand, nonlinear SVM projects both positive and negative examples into a higher-dimensional feature space and then separates them in that space. The following is a brief description of the algorithms of SVM. SVM software tools and SVM-based servers for predicting functional class of proteins and peptides are listed in Table 1.

**Figure 2.**
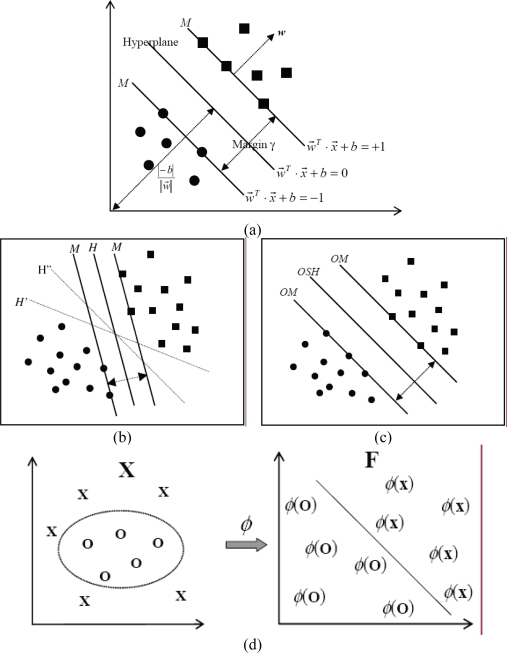
Support vector machines. (**a**) Definition of hyper-plane and margin. The circular dots and square dots represent samples of class −1 and class +1, respectively. (**b**) The available hyper-planes *H*, *H’*, *H”*, …, corresponding to a set of training data. (**c**) Unique optimal separating hyper-plane of a set of training data. (**d**) Basic idea of support vector machines: Projection of the training data nonlinearly into a higher-dimensional feature space via *φ*, and subsequent construction of a separating hyper-plane with maximum margin in that space.

Let the training data of two separate classes, each containing *n* samples, be represented by (**x**_1_, *y*_1_), (**x**_2_, *y*_2_), …, (**x***_n_*, *y_n_*), i = 1, 2, …, *n*, where **x**_i_ ∈ *R^N^* is a vector in an *N*-dimensional space representing various physicochemical and structural properties of a protein or peptide, and yi ∈ (−1, +1) indicates class label (e.g. (+) represents members and (–) non-members of a functional class). In linear SVM, given a weight vector **w** and a bias *b*, it is assumed that these two classes can be separated by two margins parallel to the hyper-plane as illustrated in Figure 2 (a), which can be represented as a single inequality:

(1) $$y_{i}(\text{w}\cdot {\text{x}}_{i}+b)\geq 1,\text{for}\;i=1,2,\ldots ,n$$

where **w** = (*w*_1,_ *w*_2_, …, *w_n_*)^T^ is a vector of *n* elements. As shown in Figure 2 (b), there are a number of separate hyper-planes for an identical group of training data. The objective of SVM is to determine the optimal weight w_0_ and optimal bias *b*_0_ such that the corresponding hyper-plane separates S+ and S– with a maximum margin and gives the best prediction performance. This hyper-plane is called Optimal Separating Hyper-plane (OSH) as illustrated in Figure 2 (c).

The equation for a hyper-plane can be written as:

(2) $$\text{w}\cdot {\text{x}}_{i}+b=0$$

By using geometry, the distance between the two corresponding margins is 2/‖*w*‖. Therefore, the OSH can be obtained by minimizing ‖**w**‖ under inequality constraints (Eq. (1)). This optimization problem could be efficiently solved with the introduction of Lagrangian multiplier *a_i_*.

(3) $$L(\text{w},b,\alpha )=\frac{1}{2}\text{w}\cdot \text{w}-\sum_{i=1}^{n}\alpha_{i}[y_{i}(\text{w}\cdot {\text{x}}_{i}+b)-1]$$

The solution to this optimization Quadratic Programming (QP) problem requires that the gradient of *L*(**w**, *b*, *α*) with respect to **w** and *b* vanishes, resulting in the following conditions:

(4) $${\text{w}}_{0}=\sum_{i=1}^{n}\alpha_{i}y_{i}{\text{x}}_{i}$$

(5) $$\sum_{i=1}^{n}\alpha_{i}y_{i}=0$$

By substituting Eqs. (4) and (5) into Eq. (3), the QP problem becomes the maximization of the following expression:

(6) $$L(\alpha )=\sum_{i=1}^{n}\alpha_{i}-\frac{1}{2}\sum_{i=1}^{n}\sum_{j=1}^{n}\alpha_{i}\alpha_{j}y_{i}y_{j}({\text{x}}_{i}\cdot {\text{x}}_{j})$$

under the constraints

(7) $$\sum_{i=1}^{n}\alpha_{i}y_{i}=0,0\leq \alpha_{i}\leq C,i=1,2,\ldots ,n$$

where *C* is a penalty for training errors for soft-margin SVM and is equal to infinity for hard-margin SVM.

The points located on the two optimal margins will have nonzero coefficients *α_i_* among the solutions to Eq. (6), and are called *Support Vectors* (SV). The bias *b*_0_ can be calculated as follows:

(8) $$b_{0}=-\frac{1}{2}\left\{\underset{\left\{{\text{x}}_{i}|y_{i}=+1\right\}}{\text{min}}({\text{w}}_{0}\cdot {\text{x}}_{i})+\underset{\left\{{\text{x}}_{i}|y_{i}=-1\right\}}{\text{max}}({\text{w}}_{0}\cdot {\text{x}}_{i})\right\}$$

After determination of support vectors and bias, the decision function that separates the two classes can be written as:

(9) $$\begin{array}{ll} f\;(\text{x}) & =\text{sign}\left[\sum_{\text{i}=1}^{n}\alpha_{i}y_{i}{\text{x}}_{i}\cdot \text{x}+b_{0}\right]\\ & =\text{sign}\left[\sum_{\text{SV}}\alpha_{i}y_{i}{\text{x}}_{i}\cdot \text{x}+b_{0}\right] \end{array}$$

Nonlinear SVM projects feature vectors into a high dimensional feature space by using a kernel function *K(x,y)*. The linear SVM procedure is then applied to the feature vectors in this feature space. After the determination of *w* and *b*, a given vector x can be classified by using

(10) $$f(x)=\text{sign}\left(\sum_{SV}\alpha_{i}y_{i}K(x,x_{i})+b_{0}\right)$$

A positive or negative value indicates that the vector *x* belongs to the members or non-members of a functional class, respectively.

In Equation (10), Kernel function *K(x,y)* represents a legitimate inner product in the input space:

(11) K(x,y)=φ(x)·φ(y)

A number of kernel functions have be used in SVM. Examples of the most popular ones are:

(12) $$\text{Polynominal}:K({\text{x}}_{i},{\text{x}}_{j})={\left({\text{x}}_{i}\cdot {\text{x}}_{j}+1\right)}^{p}$$

(13) $$\text{Gaussian}:K({\text{x}}_{i},{\text{x}}_{j})=e^{-{\left\|{\text{x}}_{j}-{\text{x}}_{i}\right\|}^{2}/2\sigma^{2}}$$

(14) $$\text{Sigmoid}:K({\text{x}}_{i},{\text{x}}_{j})=\text{tan}\;\text{h}(\kappa \;x_{i}x_{j}+c)$$

A vector has a limited number of components, each representing a specific physicochemical, structural or biological quantity. Each quantity is normalized or scaled, such that its value is of finite value. From a practical point of view, **x** · **y** is of finite value so as to avoid the value of polynomial kernel reaching infinity.

## Methods for Training, Testing and Estimating Generalization Capabilities of Support Vector Machines Classification Systems

Several validation methods have been used for training, testing, and estimating generalization errors of a SVM model (Bhasin and Raghava, 2004a; Martin et al. 2005; Plewczynski et al. 2005; Lei and Dai, 2006) based on a “re-sampling” strategy (Weiss and Kulikowski, 1991; Shao and Tu, 1995). The commonly used validation methods include N-fold cross validation, leave one out, leave v out, jack-knifing, and bootstrapping. In N-fold cross validation, samples are randomly divided into N subsets of approximately equal size. N-1 subsets are used as a training set for developing a SVM model, and the remaining one is used as a testing set for evaluating the prediction performance of that model. This process is repeated N times such that every subset is used as a testing set once. The average accuracy of the N number of SVM models is used for measuring the generalization capability of the SVM method. When N equals to the total number of samples, the method is called “leave one out” such that every sample is used for testing a SVM model trained by using all of the other samples. “Leave-v-out” is a more elaborate and expensive version of the “leave something out” cross-validation that involves leaving out all possible combinations of v samples as a test set. In jack-knifing, samples are distributed and used for training and testing the SVM models in the same way as that of “leave one out” method, but the generalization error of the derived SVM models is estimated based on the comparison of the average accuracy of subsets and that of all sets of these SVM models. In bootstrapping, different combinations of randomly selected subsets of samples are separately used for training SVM models each of which is tested by using the compounds not included in the respective training set.

Moreover, independent evaluation sets have also been used for testing the performance of SVM classification systems (Cai et al. 2003; Liu et al. 2005; Wang et al. 2005; Lin et al. 2006c). In using this approach, samples are divided into training, testing, and independent validation set based on their distribution in protein or peptide descriptor space. Protein or peptide descriptor space is defined by the commonly used structural and chemical descriptors of proteins or peptides. Samples can be clustered into groups based on their distance in the descriptor space by using such methods as hierarchical clustering (Johnson, 1967). An upper-limit of the largest separation of r can be used for restricting the size of each cluster. One or more representative samples are randomly selected from each group to form a training set that is sufficiently diverse and broadly distributed in the chemical space. One or more of the remaining compounds in each group are randomly selected to form the testing set. The remaining samples are used as the independent evaluation set, which show reasonable level of structural diversity and distinction with respect to compounds of other groups.

The performance of SVM has been measured by using the positive prediction accuracy P_+_ for proteins that have a specific property and the negative prediction accuracy P_–_ for proteins without that property (Bock and Gough, 2001; Bock and Gough, 2003; Cai and Lin, 2003; Bhasin and Raghava, 2004c; Cai et al. 2004b; Cai and Doig, 2004; Han et al. 2004b; Xue et al. 2004b; Dobson and Doig, 2005; Lo et al. 2005; Martin et al. 2005; Ben-Hur and Noble, 2005). Moreover, an overall accuracy P = (TP+TN)/N, where TP and TN is the true positive and true negative respectively and N is the number of proteins or peptides, can also be used to indicate the overall prediction performance. In some cases, P, P_+_ and P_–_ are insufficient to provide a complete assessment of the performance of a discriminative method (Provost et al. 1998; Baldi et al. 2000). Thus the Matthews correlation coefficient $MCC=(TP\times TN-FP\times FN)/\sqrt{\left(TP+FN)(TP+FP)(TN+FP)(TN+FN\right)}$ has been used for measuring the performance of support vector machine (Bhasin and Raghava 2004a; Bhasin and Raghava 2004b; Cai et al. 2004b; Han et al. 2004b; Huang et al. 2005; Kumar et al. 2006).

## Assessment of the Performance of Support Vector Machine Classification Systems

### Performance for predicting functional classes of proteins and peptides

Table 2 summarizes the reported performance of the use of SVM for predicting protein functional classes. The reported P_+_ and P_–_ values are in the range of 25.0%~100.0% and 69.0%~100.0%, with the majority concentrated in the range of 75%~95% and 80%~99.9% respectively. Based on these reported results, SVM generally shows certain level of capability for predicting the functional class of proteins and protein-protein interactions. In many of these reported studies, the prediction accuracy for the non-members appears to be better than that for the members. The higher prediction accuracy for non-members likely results from the availability of more diverse set of non-members than that of members, which enables SVM to perform a better statistical learning for recognition of non-members.

The performance of SVM for predicting functional classes of peptides are given in Table 3. Prediction of protein-binding peptides have primarily been focused on MHC-binding peptides (Bhasin and Raghava, 2004c), the reported P+ and P_–_ values for MHC binding peptides are in the range of 75.0%~99.2% and 97.5%~99.9%, with the majority concentrated in the range of 93.3%~95.0% and 99.7%~99.9% respectively. These studies have demonstrated that, apart from the prediction of protein functional classes, SVM is equally useful for predicting protein-binding peptides and small molecules.

### Performance for predicting functional classes of novel proteins

The performance of SVM for predicting the functional profile of novel proteins has also been evaluated by several studies listed in Table 4. These novel proteins are of two types. The first includes several groups of proteins that have no homologous counterpart in well-established protein database, and the second contains pairs of homologous enzymes that belong to different functional families. The non-homologous nature of the first type of novel proteins complicates the task of using sequence alignment and clustering methods for determining their functions. On the other hand, the homologous nature of the second type of novel proteins may result in false association of proteins of different functional families if sequence similarity is used as the sole indicator of functional association. Therefore, it is desirable to explore other methods with less or no reliance on homology to complement sequence similarity and clustering methods (Smith and Zhang, 1997; Eisenberg et al. 2000). From Table 4, SVM appears to have the capacity of correct prediction of 46.3%~76.7% of the novel proteins found from the literatures.

The ability of SVM in predicting the functional profile of the first type of novel proteins have been attributed to the non-discriminative nature of SVM for selecting class members, and to the use of structural and physicochemical descriptors for representing proteins (Hou et al. 2004; Han et al. 2004a; Cui et al. 2005; Han et al. 2005a; Zhang et al. 2005). In some cases, protein function is determined by specific structural and chemical features at active sites, and these features are shared by distantly related as well as closely related proteins of the same functional property (Schomburg et al. 2002). Some of these function-related features might be captured by the residue properties such as hydrophobicity, normalized van der Waals volume, polarity, polarizability, charge, surface tension, secondary structures and solvent accessibility (Bull and Breese, 1974; Lin and Timasheff, 1996), which have been incorporated in the descriptors used in the construction of the feature vectors for these proteins.

The function of a protein is determined by a variety of factors. Changes such as local active-site mutation, variations in surface loops, and recruitment of additional domains may result in functional diversity among homologous proteins (Todd et al. 2001). While these changes appear to be small at the local sequence level, some of the aspects of these changes may also be captured by the descriptors associated with hydrophobicity, normalized van der Waals volume, polarity, polarizability, charge, surface tension, secondary structure and solvent accessibility.

### Performance for predicting proteins with specific structural characteristics

Subgroups of proteins of specific functional classes are known to have common structural features. For instance, a number of RNA-binding proteins have a modular structure and contain RNA-binding domains of 70–150 amino acids that mediate RNA recognition (Mattaj, 1993; Perez-Canadillas and Varani, 2001). Three classes of RNA-binding domains have been documented to bind RNA in a sequence independent manner, and these domains are RNA-recognition motif (RRM), double-stranded RNA-binding motif (dsRM), and K-homology (KH) domain (Perez-Canadillas and Varani, 2001). A fourth class of RNA-binding domain, S1 RNA-binding domain, has also been found in a number of RNA-associated proteins (Bycroft et al. 1997). These domains have distinguished structural features responsible for RNA recognition and binding. Thus the performance of SVM classification of functional classes of proteins can be evaluated by examining whether or not proteins containing one of these domains can be correctly classified into the respective class (Han et al. 2004b; Leslie et al. 2004; Kunik et al. 2005; Lin et al. 2006c).

A search of protein family and sequence databases shows that there are a total of 260, 74, 190, and 41 RNA-binding protein sequences known to contain RRM, dsRM, KH and S1 RNA-binding domain respectively. The majority of these sequences are included in the training and testing set of all RNA-binding proteins. In the corresponding independent evaluation set, there are 35, 16, 93, and 10 sequences containing RRM, dsRM, KH, and S1 RNA-binding domain respectively. All but one protein sequence are correctly classified as RNA-binding by SVM, which shows the capability of SVM (Han et al. 2004b). The only incorrectly predicted protein sequence is HnRNP-E2 protein fragment in the group that contains KH domain. The incompleteness of this sequence might partially contribute to its incorrect prediction by SVM.

In another example, some lipid-binding proteins are known to contain lipid-binding domains or motifs (Balla, 2005). Several families of such lipid-binding proteins have been documented and examples of these families are TIM, PP-binding or GCV_H. These families have distinguished structural features responsible for lipid recognition and binding. A search of protein family and sequence databases shows that there are 227, 184, and 139 lipid-binding protein sequences known to contain TIM, PP-binding or GCV_H domain respectively. The majority of these sequences are included in the training and testing set of all lipid-binding proteins. In the corresponding independent evaluation set, there are 81, 27, and 30 sequences containing TIM, PP-binding or GCV_H domain respectively. Most of these protein sequences are correctly classified as lipid-binding by SVM, and there is only 1, 1, and 2 misclassified sequences in the TIM, PP-binding or GCV_H domain families respectively (Lin et al. 2006c). The incorrectly predicted protein sequences are triosephosphate isomerase (fragment), putative acyl carrier protein, mitochondrial precursor, glycine cleavage system H protein, mitochondrial precursor (fragment), probable glycine cleavage system H protein 2 and mitochondrial precursor. Most of these incorrectly predicted sequences are fragments. Therefore, sequence incompleteness appears to be a factor that partially contributes to the incorrect prediction of these sequences by SVM.

### Effect of different sets of protein descriptors to the classification of functional classes of proteins

As shown in Table 2 and Table 3, different sets of protein descriptors have been used in SVM prediction of various functional classes of proteins and peptides, all of which have shown impressive predictive performances (Chou and Cai, 2005; Gao et al. 2005; Li et al. 2006). Non-the-less, there is a need to comparatively evaluate the effectiveness of these descriptor-sets in a single study and to examine whether combined use of these descriptor-sets help to improve predictive performance. For such a purpose, we tested the performance of seven popular descriptor-sets and two of their combinations in SVM prediction of six different classes of proteins. These sets are amino acid composition (Chou and Cai, 2005) (class 1), dipeptide composition (Gao et al. 2005) (class 2), normalized Moreau–Broto autocorrelation (Feng and Zhang, 2000; Lin and Pan, 2001) (class 3), Moran autocorrelation (Horne, 1988) (class 4), Geary autocorrelation (Sokal and Thomson, 2006) (class 5), sets of composition, transition and distribution of physicochemical properties (Dubchak et al. 1995; Dubchak et al. 1999; Bock and Gough, 2001; Cai et al. 2003; Cai et al. 2004a; Han et al. 2004b; Lo et al. 2005; Lin et al. 2006a; Cui et al. 2007a) (class 6), sequence order (Grantham 1974; Schneider and Wrede, 1994; Chou, 2000;  Chou and Cai, 2004) (class 7), the frequently used combination of amino acid composition and dipeptide composition (Gao et al. 2005) (class 8), and combination of the seven individual sets of descriptors (class 9). The six protein functional classes are enzyme EC 2. 4 (NC - IUBMB 1992), G protein-coupled receptors, transporter TC8.A (Saier et al. 2006), chlorophyll (Suzuki et al. 1997), lipid synthesis proteins involved in lipid synthesis, and rRNA-binding proteins. These classes were selected because of their functional diversity and level of difficulty in achieving high prediction performance. The reported SVM prediction performance for these classes tend to be lower than other classes (Cai et al. 2004a), which are ideal for critically evaluating the effectiveness of different descriptor-sets.

The dataset statistics and SVM performance of the nine descriptor-sets are given in Table 5 and the overall performance scores of these descriptor-sets are given in Table 6. The overall performance scores are composed of 4 categories defined by the values of MCC of a SVM model: “Exceptional”, “Good”, “Fair” and “Poor” when MCC is in the range of >0.9, 0.8–0.9, 0.6–0.8, and <0.6 respectively. Overall, there is no single preferred descriptor-set for all cases. Sets 6, 8, and 9 tend to exhibit higher sensitivity, with the exception of chlorophyll proteins, while classes 1 and 7 tend to be among the lowest ranked. The combined classes 8 and 9 generally give the highest MCC values, again with the exception of chlorophyll proteins, while classes 1 and 7 tend to return the lowest MCC values. These findings are consistent with the results from a reported study that suggest that amino acid composition, polarity, solvent accessibility and charge, are more important than other properties, in order of prominence, for SVM classification of specific protein functional classes (Lin et al. 2006b). Using the entire set of descriptors (class 9) does not necessarily always gives better performance, which is consistent with the findings that analysis of the contribution of individual descriptors and the selection of the relevant ones are highly useful for improving SVM prediction performance (Glen et al. 1989; Xue et al. 1999; Xue and Bajorath 2000; Xue et al. 2000).

### Contribution of individual protein descriptors to the classification of functional classes of proteins

In using SVM for predicting functional classes of proteins, several descriptors have been used to describe physicochemical characteristics of each protein (Bock and Gough, 2001; Ding and Dubchak, 2001; Cai et al. 2002a; Cai et al. 2002b; Cai et al. 2003; Han et al. 2004b). It has been reported that, not all descriptors contribute equally to the classification of proteins, some have been found to play relatively more prominent role than others in specific aspects of proteins (Ding and Dubchak, 2001). It is therefore of interest to examine which descriptors are more important in the classification of proteins. Contribution of individual descriptors to protein classification has been investigated by separately conducting classification using each feature property (Ding and Dubchak, 2001). By using the same method, one finds that, in order of prominence, the polarity, hydrophobicity, amino acid composition, and solvent accessibility play more prominent roles than other feature properties in the classification of lipid-binding protein (Lin et al. 2006c). Polarity and hydrophobicity have been shown to be important for lipid-protein interactions such that lipid binding sites are located in a hydrophobic and low polarity environment (Lugo and Sharom, 2005). High-affinity lipid binding site in some proteins appear to be located at sequence segments with specific amino acid composition (Hamilton et al. 1986), and specific sequence motifs have been used for predicting lipid-binding proteins (Gonnet and Lisacek, 2002; Eisenhaber et al. 2003; Juncker et al. 2003; Gonnet et al. 2004; Eisenhaber et al. 2004). A study of apolipophorin-III in lipid-free and phospholipid-bound states showed that lipid-binding involves increased solvent accessibility due to gross tertiary structural reorganization (Raussens et al. 1996). Therefore, the selected descriptors are consistent with these experimental findings.

### Analysis of descriptor contributions by using feature selection method

More rigorous feature selection methods (Xue et al. 2004a; Al-Shahib et al. 2005a; Al-Shahib et al. 2005b;), such as recursive feature elimination (RFE) (Guyon et al. 2002), can be applied to the SVM classification of functional classes of proteins to select those descriptors most relevant to the prediction of proteins of a particular class (Guyon et al. 2002; Yu et al. 2003). The details of the implementation of this method can be found in the literatures (Xue et al. 2004a; Xue et al. 2004b). Feature selection procedure can be demonstrated by the following illustrative example of the development of a SVM classification system for predicting DNA-binding proteins: This system is trained by using a Gaussian kernel function with an adjustable parameter σ. Sequential variation of σ is conducted against the whole training set to find a value that gives the best prediction accuracy. This prediction accuracy is evaluated by means of 5-fold cross-validation. In the first step, for a fixed σ, the SVM classifier is trained by using the complete set of features (protein descriptors) described in the previous section. The second step involves the computation of the ranking criterion score DJ(i) for each feature in the current set. All of the computed DJ(i) is subsequently ranked in descending order. The third step involves the removal the m features with smallest criterion scores. In the fourth step, the SVM classification system is re-trained by using the remaining set of features, and the corresponding prediction accuracy is computed by means of 5-fold cross-validation. The first to fourth steps are then repeated for other values of σ. After the completion of these procedures, the set of features and parameter σ that give the best prediction accuracy are selected.

A total of 28 features were selected by RFE, which are given in Table 7. In order of prominence, compositions of specific amino acids, Van der Waalse volume, polarity, polarizability, surface tension, secondary structure, and solvent accessibility are found to be important for predicting DNA-binding proteins. Protein-DNA binding is known to involve specific recognition sequence and induced conformation changes (Cheng et al. 1993). Therefore it is expected that the combined features of amino acid composition and surface tension is important for characterizing DNA-binding proteins. DNA binding also involves spatial arrangement or pre-arrangement of specific group of amino acids at the binding site (Patel et al. 2006). It is thus not surprising that such important interactions as polarizability, hydrophobicity, polarity and surface tension are coupled to the size of the amino acid sequence segment at a DNA-binding site. Many proteins bind DNA via minor groove interaction between protein non-polar surfaces and DNA hydrophobic sugar clusters (Tolstorukov et al. 2004). As a result, the combined features of hydrophobicity and solvent accessibility are expected to be important for describing these proteins.

The usefulness of these 28 selected features can be further tested by constructing a SVM classification system based solely on these features. The prediction accuracies of this new system are 87.2% and 92.6% for DNA-binding and non-DNA-binding proteins respectively, which is slightly improved against those of 85.7% and 91.2% by using all features. This suggests that the use of selected subset of features enhances prediction performance by reducing the noise created by the redundant and irrelevant features.

### Comparison of SVM prediction performance under different kernel functions

Apart from the Gaussian kernel function of sequence-derived physicochemical properties, several other kernel functions have been developed and applied for SVM classification of proteins and DNAs (Jaakkola et al. 1999; Zien et al. 2000; Tsuda et al. 2002; Vert et al. 2003; Vishwanathan and Smola, 2003; Leslie et al. 2003; Liao and Noble, 2003; Ratsch et al. 2005; Kuang et al. 2005). It is of interest to test the usefulness of some of these kernel functions for predicting functional classes of proteins. The string-kernel function has been extensively used and it has shown promising potential for protein and DNA studies (Vishwanathan and Smola, 2003; Ratsch et al. 2005). This kernel function is constructed by comparison of sequences of classes of proteins or DNAs and the assignment of individual weights to amino acids or nucleotides to describe physicochemical or other characteristics of the proteins and DNAs. This kernel function is used to develop three SVM systems for predicting the class of lipid-degradation, lipid metabolism, and lipid synthesis proteins. Spectrum kernel with mismatches (Leslie et al. 2003) is used to generate the string-kernel for each protein. Testing results by using an independent set of proteins for each class show that the SE is 77.2%, 75.8%, 77.8%, and the SP is 97.6%, 96.4%, 94.2% for each of these classes respectively (Lin et al. 2006c). Thus comparable prediction performance can be achieved by using string-kernel SVM, which suggests the usefulness of this and other kernel functions for SVM prediction of functional classes of proteins.

### Comparison of SVM prediction performance with other machine learning methods

Several other machine learning (ML) methods have been explored for predicting the functional classes of proteins and peptides. These methods include artificial neural network (ANN), k-nearest neighbors (KNN), decision tree and hidden Markov model (HMM). They have been used for predicting enzymes (Jensen et al. 2002), receptors (Jensen et al. 2003), transporters (Jensen et al. 2003), structural proteins (Jensen et al. 2003), mitochondrial proteins (Kumar et al. 2006), cell cycle regulated proteins (de Lichtenberg et al. 2003), growth factors (Jensen et al. 2003), and allergen proteins (Zorzet et al. 2002; Soeria-Atmadja et al. 2004). The reported P+ and P– values of these ML methods are in the range of 37.8%~87% and 66.0%~99.9%, with the majority concentrated in the range of 60%~85% and 70%~90% respectively. These values are slightly lower than the values of 75%~95% and 80%~99.9% of the SVM, suggesting that other ML methods are also useful for predicting the functional class of proteins and peptides.

## Underlying Difficulties in Using Support Vector Machines

The performance of SVM critically depends on the diversity of samples (proteins and peptides) in a training dataset and the appropriate representation of these samples. The datasets used in many of the reported studies are not expected to be fully representative of all of the proteins, peptides and small molecules with and without a particular functional and interaction profile. Various degrees of inadequate sampling representation likely affect, to a certain extent, the prediction accuracy of the developed statistical learning models. SVM is not applicable for proteins, peptides and small molecules with insufficient knowledge about their specific functional and interaction profile. Searching of the information about proteins, peptides and small molecules known to possess a particular profile and those do not possess that profile is a key to more extensive exploration of statistical learning methods for facilitating the study of protein functional and interaction profiles. Apart from literature sources such as PubMed (Beebe, 2006), databases such as Swiss-Prot (Dorazilova and Vedralova, 1992), Genbank (Benson et al. 2004), pirpsd (Barker et al. 1999), geneontology (Chalmel et al. 2005), PDB (Berman et al. 2000), enzyme database (Bairoch, 2000), TransportDB (Ren et al. 2004), HMTD (Yan and Sadee, 2000), ABCdb (Quentin and Fichant, 2000), TiPS (Alexander, 1999), GPCRDB (Horn et al. 2003), SYFPEITHI (Rammensee et al. 1999), MHCPEP (Brusic et al. 1996), JenPep (Blythe et al. 2002), MHCBN (Bhasin et al. 2003), FIMM (Schonbach et al. 2000), and FSSP database (Holm and Sander, 1996) are also useful for obtaining information about protein/peptide functional and interaction profiles.

In the datasets of some of the reported studies, there appears to be an imbalance between the number of samples having a profile and those without the profile. SVM method tends to produce feature vectors that push the hyper-plane towards the side with smaller number of data (Veropoulos, 1999), which often lead to a reduced prediction accuracy for the class with a smaller number of samples or less diversity than those of the other class. It is however inappropriate to simply reduce the size of non-members to artificially match that of members, since this compromises the diversity needed to fully represent all non-members. Computational methods for re-adjusting biased shift of hyperplane are being explored (Brown et al. 2000). Application of these methods may help improving the prediction accuracy of SVM in the cases involving imbalanced data.

While a number of descriptors have been introduced for representing proteins and peptides (Bock and Gough, 2001; Karchin et al. 2002; Cai et al. 2003; Gasteiger, 2005), most reported studies typically use only a portion of these descriptors. It has been found that, in some cases, selection of a proper subset of descriptors is useful for improving the performance of SVM (Xue et al. 2004a; Al-Shahib et al. 2005a; Al-Shahib et al. 2005b). Therefore, there is a need to explore different combination of descriptors and to select more optimum set of descriptors for more cases, which can be conducted by using feature selection methods (Xue et al. 2004a; Al-Shahib et al. 2005a; Al-Shahib et al. 2005b). Efforts have also been directed at the improvement of the efficiency and speed of feature selection methods (Furlanello et al. 2003), which will enable a more extensive application of feature selection methods. Moreover, indiscriminate use of the existing descriptors, particularly those of overlapping and redundant descriptors, may introduce noise as well as extending the coverage of some aspects of these special features. Thus, it may be necessary to introduce new descriptors for the systems that have been described by overlapping and redundant descriptors. Investigation of cases of incorrectly predicted samples have also suggested that the currently-used descriptors may not always be sufficient for fully representing the structural and physicochemical properties of proteins, peptides and small molecules (Xue et al. 2004b; Li et al. 2005; Yap and Chen, 2005). These have prompted works for developing new descriptors (Bhardwaj et al. 2005).

## Concluding remarks

SVM has consistently shown promising capability for predicting functional classes of proteins and peptides. Proper use of descriptors for representing proteins and peptides may help further improving the performance of SVM for predicting functional profiles of proteins and peptides. The introduction of new descriptors would better represent characteristics that correlate with novel functional and interaction profiles. Moreover, various feature selection methods may be used for selecting optimal set of descriptors for a particular prediction problem. Existing algorithms can be improved and new algorithms may be introduced for enhancing the performance and accuracy of support vector machine. The prediction capability of SVM can be further enhanced with increasing availability of biological data and more extensive knowledge about sequence, structure, transcription, post-transcriptional processing features that define the functional profiles of proteins and peptides. These efforts will enable the development of SVM into useful tools for facilitating the study of functional profiles of proteins and peptides to complement other well-established methods such as sequence similarity and clustering methods.

## Figures and Tables

**Table 1.** Web-servers for computing functional class of proteins and peptides by using support vector machines. Web-sites of support vector machine software are also given.

**Category**	**Web-server or software**	**URL**
Server for Predicting Protein Functional Class	CTKPred: SVM prediction and classification of the cytokine family	http://bioinfo.tsinghua.edu.cn/~huangni/CTKPred/
	GPCRpred: SVM prediction of families and subfamilies of G-protein coupled receptors	http://www.imtech.res.in/raghava/gpcrpred/info.html
	pSLIP: SVM protein subcellular localization prediction	http://pslip.bii.a-star.edu.sg/
	SVMProt: SVM protein functional family prediction from protein sequence	http://jing.cz3.nus.edu.sg/cgi-bin/svmprot.cgi
Server for Predicting Peptide Functional Class	MHC-BPS: SVM prediction of MHC-binding peptides of flexible lengths	http://bidd.cz3.nus.edu.sg/mhc/
	SVMHC: SVM prediction of MHC-binding peptides	http://www.sbc.su.se/svmhc/
	SVRMHC: SVM prediction of MHC-binding peptide	http://svrmhc.umn.edu/SVRMHCdb/
	WAPP: SVM prediction of MHC-binding, proteasomal cleavage and TAP transport peptides	http://www-bs.informatik.unituebingen.de/WAPP
SVM Software and servers	SVM light	http://svmlight.joachims.org/
	LIBSVM	http://www.csie.ntu.edu.tw/~cjlin/libsvm/
	mySVM	http://www-ai.cs.unidortmund.de/SOFTWARE/MYSVM/index.html
	SMO	http://www.datalab.uci.edu/people/xge/svm/
	BSVM	http://www.csie.ntu.edu.tw/~cjlin/bsvm/
	WinSVM	http://www.cs.ucl.ac.uk/staff/M.Sewel1/winsvm/
	LS-SVMlab	http://www.esat.kuleuven.ac.be/sista/lssvmblab/
	GIST SVM Server	http://svm.sdsc.edu

**Table 2.** Performance of machine learning methods for predicting functional class of proteins as reported in the literature. All of the data and results were collected from the original papers. Please refer to the respective references for complete results. N+, N– and N are the number of class members, non-members and all proteins (members + non-members) respectively, P+ and P– are prediction accuracy for class members and non-members respectively, P is the overall accuracy, and MCC is the Matthews correlation coefficient.

**Protein functional class**	**Protein Sub-classes**	**Protein descriptors**	**Number of proteins in training Set N (N+/N–)**	**Validation method**	**Reported prediction accuracy**				**Ref**
					**P+ (%)**	**P– (%)**	**P (%)**	**MCC**	
Enzymes	46 sub-classes: EC1.1~EC1.11, EC1.13~EC1.15, EC1.17, EC1.18, EC2.1~EC2.8, EC3.1~EC3.6, EC4.1~EC4.4, EC4.6, EC5.1~EC5.5, EC5.99, EC6.1~EC6.5	Physicochemical properties	956~9216 (35~3892/807~5324)	Independent evaluation	53.0~ 99.3	85.0~ 99.7	81.8~ 99.7	0.31 ~ 0.98	(Cai et al 2003; Cai et al. 2004b)
	54 sub-classes: EC1.1~EC1.21, EC2.1~EC2.8, EC3.1~EC3.8, EC4.1~EC4.6, EC5.1~Ec5.6, EC6.1~6.6	Functional Domain Composition and pseudo amino acid composition	503~3582 (3~2002/327~3548)	Jackknife Test	25.0~ 100.0				(Cai and Chou, 2005)
Transporters	20 sub-classes: TC1.A, TC1.A.1, TC1.B, TC1.E, TC2.A, TC2.A.1, TC2.A.3, TC2.A.6, TC2.C, TC3.A, TC3.A.1, TC3.A.3, TC3.A.5, TC3.A.15, TC3.D, TC3.E, TC4.A, TC8.A, TC9.A, TC9.B	Physicochemical properties	613~7508 (50~1220/513~7299)	Independent evaluation	60.6~ 97.1	91.5~ 99.9	91.4~ 99.7	0.27~ 0.97	(Lin et al. 2006a)
Allergenic proteins		Amino acid	1278 (578/700)	Independent evaluation	88.9	81.9	85.0	0.71	(Saha and Raghava, 2006)
		Dipeptide composition	1278 (578/700)	Independent evaluation	82.8	85.0	84.0	0.68	
		Physicochemical properties	23474 (1005/22469)	Independent evaluation	93.0	99.9	99.7	0.96	(Cui et al. 2007b)
Crystallizable proteins		Mono-, di-, tri-peptide composition, physicochemical and structural properties	923 (721/202)	10-fold CV	65.0	69.0	67.0		(Smialowski et al. 2006)
Mitochondrial proteins		Amino acid composition	10372 (1432/8940)	5-fold CV	78.9	90.0	88.2	0.62	(Kumar et al. 2006)
G-protein coupled receptors	All GPCRs	Physicochemical properties	2247 (927/1320)	Independent evaluation	95.6	98.1	97.4	0.93	(Cai et al. 2003)
		Dipeptide composition	3302 (778/2524)	5-fold CV	98.6	99.8	99.5	0.99	(Bhasin and Raghava, 2004b)
		Protein power spectrum	946	Jackknife			96.1		(Guo et al. 2006)
	Gi/o binding type	Structural characteristics	132 (61/71)	4-fold CV	77.0	78.3			(Yabuki et al. 2005)
	Gq/11 binding type	(extra cellular loops, intracellular loops etc)	132 (47/85)	4-fold CV	68.1	72.7			
	Gs binding type		132 (24/108)	4-fold CV	83.3	95.2			
	Rhodopsin-like (Class A)	Protein power spectrum	540	Jackknife			97.0	0.93	(Guo et al. 2006)
	Secretin-like (Class B)		187	Jackknife			96.3	0.94	
	Metabotropic glutamate (Class C)		103	Jackknife			94.2	0.95	
	Fungal pheromone (Class D)		21	Jackknife			81.0	0.92	
	cAMP receptors (Class E)		5	Jackknife			100.0	1	
	Frizzled/smoothened (Class F)		90	Jackknife			95.6	0.94	
Nuclear receptors	All nuclear receptors	Amino acid composition	282	5-fold CV			82.6	0.74	(Bhasin and Raghava,
		Dipeptide composition	282	5-fold CV			97.5	0.96	2004a)
		Physicochemical properties	872 (334/538)	Independent evaluation	89.5	97.6			(Cai et al. 2003)
		Protein power spectrum	465	Jackknife			95.3		(Guo et al. 2006)
	Thyroid hormone-like	Protein power spectrum	165	Jackknife			95.8	0.95	(Guo et al. 2006)
	HNF4-like		114	Jackknife			97.4	0.96	
	Estrogen-like		130	Jackknife			97.7	0.96	
	Fushitarazu-F1 like		35	Jackknife			94.3	0.97	
	Nerve growth factor IB-like		5	Jackknife			80.0	0.89	
	Germ cell nuclear receptor		2	Jackknife			100.0	1.0	
	0A Knirps-like		7	Jackknife			42.9	0.65	
	0B DAX-like		7	Jackknife			71.4	0.84	
RNA-binding proteins	All RNA-binding proteins	Amino acid composition and limited range correlation of hydrophobicity and solvent accessible surface area	6264 (1496/4768)	10-fold CV	76.5	97.2	92.2		(Cai and Lin, 2003)
		Physicochemical properties	5126 (2161/2965)	Independent evaluation	97.8	96.0	96.1	0.8	(Han et al. 2004b)
	rRNA-binding	Amino acid composition, limited range correlation of hydrophobicit, solvent accessible surface area	5824 (1056/4768)	10-fold CV	100.0	99.9	99.9		(Cai and Lin, 2003)
		Physicochemical properties	1680 (708/972)	Independent evaluation	94.1	98.7	98.6	0.74	(Han et al. 2004b)
	tRNA-binding	Physicochemical properties	886 (94/792)	Independent evaluation	94.1	99.9	99.8	0.92	(Han et al. 2004b)
	mRNA-binding		2383 (277/2106)		79.3	96.5	96.0	0.53	
	snRNA-binding		2021 (33/1988)		45.0	99.7	99.5	0.38	
DNA-binding proteins	All DNA-binding proteins	Amino acid composition, limited range correlation of hydrophobicity, solvent accessible surface area	12507 (7739/4768)	10-fold CV	92.8	77.1	86.8		(Cai and Lin, 2003)
		Surface and overall composition, overall charge and positive potential patches on the protein surface	359 (121/238)	5-fold CV	89.1	82.1	93.9		(Bhardwaj et al. 2005)
				Jackknife	90.5	81.8	94.9		
				leave 1-pair holdout	86.3	80.6	87.5		
				Leave-half holdout	83.3	82.5	83.5		
		Physicochemical properties	8575 (4240/4335)	Independent evaluation	90.9	87.6	88.5	0.74	(Cai et al. 2003; Lin et al. 2006b)
	DNA condensation	Physicochemical properties	2410 (50/2360)	Independent evaluation	94.9	98.3	98.3	0.47	(Cai et al. 2003; Lin et al. 2006b)
	DNA integration		1307 (134/1173)		87.9	99.9	99.7	0.91	
	DNA recombination		3357 (889/2468)		87.8	98.9	97.9	0.87	
	DNA repair		5785 (2142/3643)		88.7	96.8	95.3	0.84	
	DNA replication		3734 (1131/2603)		85.6	96.6	95.4	0.79	
	DNA-directed		2348 (273/2075)		72.9	99.7	98.9	0.79	
	DNA polymerase								
	DNA-directed		2594 (484/2110)		90.8	99.4	98.8	0.91	
	RNA polymerase								
	Repressor		3684 (1337/2347)		93.3	95.6	95.4	0.76	
	Transcription factors		2354 (670/1684)		86.1	99.5	99.3	0.79	
Lipid-binding proteins	All lipid-binding proteins	Physicochemical properties	6933 (3232/3701)	Independent evaluation	89.9	97	94.1	0.88	(Cai et al. 2003; Lin et al. 2006c)
	Lipid transport		2262 (153/2109)		79.5	99.8	99.6	0.8	
	Lipid metabolism		2262 (293/1969)		79.5	99.2	98.8	0.72	
	Lipid synthesis		3498 (891/2607)		82.2	99.6	98.1	0.87	
	Lipid degradation		2178 (403/1775)		78.9	99.9	99.3	0.87	
Transmembrane proteins	Functional Domain Composition		2059	jackknife test			86.3		(Cai et al. 2003)
				independent test			67.5		
				self-consistency			93.9		
		Pseudo-amino acid composition	2059	jackknife test			82.4		(Wang et al. 2004)
				independent test			90.3		
				self-consistency			99.9		
		Physicochemical properties	4668 (2105/2563)	Independent evaluation	90.1	86.7	86.7	0.75	(Cai et al. 2003)
Cytokines	All cytokines	Dipeptide composition	1110 (437/673)	7-fold CV	92.5	97.2	95.3	0.9	(Huang et al. 2005)
	FGF/HBGF		437 (83/354)		92.7	98.6	97.5	0.92	
	TGF-β		437 (190/247)		97.4	94.7	95.8	0.92	
	TNF		437 (96/341)		94.0	98.8	97.7	0.94	
	Joint class (IL-6, LIF//OSM, MDK/PTN, NGF)		437 (68/369)		91.0	99.7	98.4	0.94	
	6 sub-classes: BMP, GDF, GDNF, INH, TGFB, other		N.A		46.7~ 100	85.5~ 100	84~ 98	0.65~ 0.96	
Functional classes in yeast	All proteins 13 classes: Metabolism, energy, cell growth, cell division, DNA synthesis, transcription, protein synthesis, protein destination, transport facilitation, intra-cellar transport, cellular biogenesis, signal transduction, cell rescue, ionic homeostasis, cellular organization	Functional domain composition	4902	Jackknife	72.0	(Cai and Doig, 2004)			
			86~725	Jackknife	15~90				

**Table 3.** Performance of support vector machine prediction of functional classes of peptides. N+ and N– are the number of members and non-members in a class, P+ and P– are the reported prediction accuracy for members and non-member respectively, and P is the reported overall accuracy.

**HLA Allele**	**Peptide descriptors**	**Number of peptides in training set N (N+/N–)**	**Validation method (N+/N–)**	**Reported prediction accuracy**			**Reference**
				**P_+_(%)**	**P_−_(%)**	**P(%)**	
A0201	Orthogonal factors from physical properties	(36/167)	10-fold cross validation	76.3	71.2	71.6	(Zhao et al. 2003)
				55.0	87.4	81.7	
				46.3	89.8	86.7	
	Amino acid sequence	113	10-fold cross validation	90.0		78.0 (Mc)	(Donnes and Elofsson, 2002)
	physico-chemical properties	(1125/6911)	Validationset (130/6664)	99.2	97.5	97.5	
A1	Amino acid sequence	28	10-fold cross validation	98.0		96.0 (Mc)	(Donnes and Elofsson, 2002)
	physico-chemical properties	(200/6831)	Validation set (40/6830)	75.0	99.7	99.6	
A3	Amino acid sequence	73	10-fold cross validation	91.0		80.0 (Mc)	(Donnes and Elofsson, 2002)
	physico-chemical properties	(139/6833)	Validation set (30/6833)	93.3	98.8	98.7	
B8	Amino acid sequence	25	10-fold cross validation	91.0		79.0 (Mc)	(Donnes and Elofsson, 2002)
	physico-chemical properties	(168/6833)	Validation set (20/6830)	95.0	99.8	99.8	
B2705	Amino acid sequence	29	10-fold cross validation	100.0		100.0 (Mc)	(Donnes and Elofsson, 2002)
	physico-chemical properties	(141/7361)	Validation set (21/7359)	95.0	99.9	99.9	
DRB1.0401	Binary code of amino acid sequence	567	5-fold cross validation	80.287.1	77.485.0	78.886.1	(Bhasin and Raghava, 2004d)
	physico-chemical properties	(539/6883)	Validation set (100/6704)	95.0	99.9	99.9	

**Table 4.** Performance of support vector machine prediction of functional classes of novel proteins.

**Protein group and year of report**	**No. of proteins or protein pairs**	**Percentage of correctly predicted proteins**	**Examples of correctly predicted proteins or protein pairs**	**Examples of incorrectly predicted proteins or protein pairs**
Enzymes without a homolog in NR databases 2004 (Han et al. 2004a)	12	66.7%	Thiocyanate hydrolase beta subunit (EC 3.5.5.8) [O66186]	Extracellular phospholipase (EC 3.1.1.5) [P82476]
			Potential cysteine protease avirulence protein avrPpiC2 (EC 3.4.22.-) [Q9F3T4]	Alginate lyase precursor (EC4.2.2.3) [P39049]
			Extracellular phospholipase (EC 3.1.1.5) [P82476]	
Enzymes without a homolog in Swissprot database 2004 (Han et al. 2004a)	50	72%	DNA polymerase III, theta subunit (EC 2.7.7.7) [P28689]	Beta-agarase B (EC 3.2.1.81) [P488401] Alpha-N-AFase II (EC 3.2.1.55) [P39049]
			Telomere elongation protein (EC2.7.7.-) [P17214]	
			Ammonia monooxygenase (EC 1.13.12.-) [Q04508]	
Viral proteins without a homolog in Swissprot database 2004 (Han et al. 2005a)	25	72%	Endonuclease II[P07059] Outer capsid protein VP4 [P35746]	TRL10 (Structural envelop glycoprotein) [AAL27474]
			Protein kinase [P00513]	BARF0 protein [Q8AZJ4]
Bacterial proteins without a homolog in Swissprot database 2004 (Cui et al. 2005)	90	76.7%	2-aminomuconate deaminase [P81593]	Alginate lyase [Q59478]
			Aminopeptidase G [Q54340]	Alpha-N-AFase II [P82594]
Plant proteins without a homolog in Swissprot database (Han et al. 2005b)	31	71.4%	Antimicrobial peptide 4 [AAL05055]	LeMan3 [Q9FUQ6] MAN5 [Q6YM50]
			Sucrose phosphatase [Q84ZX9]	
Pairs of homologous enzymes of different families 2004 (Han et al. 2004a)	8	62%	Glycolateoxidase [P05414] and IPP isomerase [Q84W37] Creatine amidinohydrolase [P38488] and Prolinedipeptidase [O58885]	Cystathionine gamma-synthase [P38675] and Methionine gamma-lyase [P13254]
				Exocellobiohydrolase 1[P38676] and Cystathionine gamma-lyase [Q8VCN5]
Remote homologs (Zhang et al. 2005) from FSSP database (Holm and Sander, 1996) 2005	445	46.3%	1cem (1,4-D-glucan-glucanohydrolase catalytic domain) and it’s remote homolog 1qazA (Alginate lyase A1–III from Sphingomonas Species; Chain: A;)	

**Table 5.** Dataset statistics and prediction performance of SVM prediction of six protein functional classes by using different descriptor sets

Protein functional family	Descriptor class	Trainingset		Testing set				Independent evaluation set						Q(%)	MCC
		P	N	P		N		P			N				
				TP	FN	TN	FP	TP	FN	Sen(%)	TN	FP	Spec(%)		
EC2.4	1	1249	2120	1154	1	9065	12	724	176	80.4	5064	4	99.9	97.0	0.879
	2	1319	2120	1080	5	8806	1	646	154	82.9	5067	1	100.0	97.4	0.884
	3	1105	1756	1295	4	9166	5	768	132	85.3	5066	2	100.0	97.8	0.911
	4	1239	2221	1161	4	8701	5	756	144	84.0	5067	1	100.0	97.6	0.903
	5	1242	2223	1160	2	8690	14	753	147	83.7	5065	3	99.9	97.5	0.900
	6	1214	2077	1145	45	8846	4	741	159	82.3	5067	1	100.0	97.3	0.893
	7	1293	2624	1072	39	8295	8	696	204	77.3	5065	3	99.9	96.5	0.860
	8	1275	2747	1129	0	8177	3	782	118	86.9	5965	3	99.9	98.0	0.921
	9	1358	3887	1015	31	7040	0	796	104	88.4	5067	1	100.0	98.2	0.930
GPCR	1	1590	7458	1847	1	14166	3	501	12	97.7	6776	62	99.1	99.0	0.927
	2	564	711	1728	3	14121	5	498	15	97.1	6800	38	99.4	99.3	0.946
	3	1169	4628	1122	4	10208	1	491	22	95.7	6800	38	99.4	99.2	0.938
	4	1257	4474	1037	1	10363	0	492	21	95.9	6790	48	99.3	99.1	0.930
	5	1290	4724	997	8	10113	0	487	26	94.9	6795	43	99.4	99.1	0.929
	6	757	2060	1536	2	12777	0	494	19	96.3	6813	25	99.6	99.4	0.951
	7	812	2950	1482	1	11887	0	487	26	94.9	6746	92	98.7	98.4	0.885
	8	1590	7458	693	12	7322	57	503	10	98.1	6780	58	99.2	99.1	0.933
	9	834	4361	1461	0	10476	0	493	20	96.1	6819	19	99.7	99.5	0.959
TC8.A	1	98	8014	9	0	13105	0	17	46	27.0	7962	0	100.0	99.4	0.518
	2	94	7962	50	0	14824	0	41	22	65.1	7962	0	100.0	99.7	0.806
	3	94	7962	53	0	14501	0	42	21	66.7	7962	0	100.0	99.7	0.815
	4	94	7962	47	0	11250	0	37	26	58.7	7962	0	100.0	99.7	0.765
	5	94	7962	47	0	11137	0	37	26	58.7	7962	0	100.0	99.7	0.765
	6	94	7962	64	0	15283	0	44	19	69.8	7962	0	100.0	99.8	0.835
	7	94	7962	59	0	15045	0	43	20	68.3	7962	0	100.0	99.8	0.825
	8	114	810	52	0	15114	0	41	22	65.1	7962	0	100.0	99.7	0.806
	9	103	1077	63	0	14847	0	47	16	74.6	16	0	100.0	99.8	0.863
Chlorophyll	1	523	1559	166	0	14297	0	70	12	85.4	6830	16	99.8	99.6	0.83
	2	440	934	248	1	7927	1	73	9	89.0	6841	5	99.9	99.8	0.91
	3	425	603	264	0	15253	0	77	5	93.9	6841	5	99.9	99.9	0.94
	4	415	574	273	1	15282	0	75	7	91.5	6842	4	99.9	99.8	0.93
	5	429	615	259	1	15240	1	75	7	91.5	6843	3	100.0	99.9	0.94
	6	482	946	202	5	14910	0	72	10	87.8	6844	2	100.0	99.8	0.92
	7	394	3337	210	85	12517	2	62	20	75.6	6834	12	99.8	99.5	0.79
	8	399	1273	289	1	14582	1	77	5	93.9	6832	14	99.8	99.7	0.89
	9	458	477	231	0	15379	0	76	6	92.7	6842	4	99.9	99.9	0.93
Lipid synthesis	1	849	2026	705	3	8229	7	476	159	75.0	5882	4	99.9	97.5	0.850
	2	927	2037	629	1	8225	0	507	128	79.8	5886	0	100.0	98.0	0.884
	3	898	2968	659	0	7294	0	509	126	80.2	5886	0	100.0	98.1	0.886
	4	968	3227	588	1	7035	0	493	142	77.6	5886	0	100.0	97.8	0.871
	5	970	3280	586	1	6982	0	491	144	77.3	5886	0	100.0	97.8	0.869
	6	874	2112	681	2	8149	1	525	110	82.7	5884	2	100.0	98.3	0.899
	7	863	2415	692	2	7845	2	512	123	80.6	5883	3	100.0	98.1	0.886
	8	815	1613	740	2	8638	11	525	110	80.7	5879	7	99.9	98.2	0.961
	9	800	3492	757	0	6770	0	541	94	85.2	5886	0	100.0	98.6	0.916
rRNA binding	1	548	579	3390	6	9598	22	1821	90	95.3	4662	6	99.9	98.5	0.964
	2	1133	1225	2811	0	8974	0	1827	84	95.6	4668	0	100.0	98.7	0.969
	3	1126	1638	2816	2	8560	1	1811	100	94.8	4668	0	100.0	98.5	0.963
	4	1337	1958	2697	0	8241	0	1783	128	93.3	4668	0	100.0	98.1	0.953
	5	1372	1976	2572	0	8223	0	1784	127	93.4	4668	0	100.0	98.1	0.953
	6	921	1208	2971	52	8991	0	1824	87	95.5	4668	0	100.0	98.7	0.968
	7	878	2743	3040	26	7442	14	1808	103	97.9	4634	34	99.3	97.9	0.951
	8	810	972	3075	3	9182	2	1848	63	96.7	4668	0	100.0	99.0	0.977
	9	1103	3175	2815	26	7024	0	1805	106	94.5	4668	0	100.0	98.4	0.961

**Table 6.** MCC-based performance scores of SVM prediction of different protein functional classes by using different descriptor classes.

**Protein functional class**	**Exceptional** > 0.9	**Good** 0.8–0.9	**Fair** 0.6–0.8	**Poor** < 0.6
EC2.4	9, 8, 3, 4, 5	6, 2, 1, 7		
GPCR	9, 6, 2, 3, 8, 4, 5, 1	7		
TC8.A		9, 6, 7, 3, 2, 8	4, 5	1
Chlorophyll	3, 5, 4, 9, 6, 2	8, 1	7	
Lipid synthesis	8, 9	6, 7, 3, 2, 4, 5, 1		
rRNA binding	8, 2, 6, 1, 3, 9, 5, 4, 7			

**Table 7.** Protein descriptors important for characterizing DNA-binding proteins as selected by a feature selection method, recursive feature elimination method.

**Descriptor ranking**	**Descriptor index**	**Structural or physicochemical property of descriptor**
1	F168	Solvent accessibility Composition Group 1
2	F166	Secondary structure Group 3 3/4th Distribution
3	F147	Secondary structure Composition Group 1
4	F75	Polarity Group 2 1/4th First Distribution
5	F43	Normalized Van der Waals volume Composition Group 2
6	F155	Secondary structure Group 1 2/4th Distribution
7	F91	Polarizability Group 1 1/4th First Distribution
8	F143	Surface tension Group 3 1/4th First Distribution
9	F171	Solvent accessibility Transition Group 1
10	F126	Surface tension Composition Group 1
11	F87	Polarizability Transition Group 1
12	F145	Surface tension Group 3 3/4th Distribution
13	F15	Composition of R
14	F6	Composition of G
15	F177	Solvent accessibility Group 1 3/4th Distribution
16	F154	Secondary structure Group 1 1/4th First Distribution
17	F89	Polarizability Transition Group 3
18	F133	Surface tension Group 1 1/4th First Distribution
19	F42	Normalized Van der Waals volume Composition Group 1
20	F85	Polarizability Composition Group 2
21	F175	Solvent accessibility Group 1 1/4th First Distribution
22	F130	Surface tension Transition Group 2
23	F127	Surface tension Composition Group 2
24	F151	Secondary structure Transition Group 2
25	F98	Polarizability Group 2 3/4th Distribution
26	F8	Composition of I
27	F67	Polarity Transition Group 2
28	F148	Secondary structure Composition Group 2
